# Screen Time, Sitting Time, Physical Activity, and Multisite Musculoskeletal Pain in University Students: Implications for Preventive Healthcare

**DOI:** 10.3390/healthcare14142154

**Published:** 2026-07-17

**Authors:** Elif Esma Bayraktar, Mehrad Mohammadzadeh

**Affiliations:** 1 Department of Physiotherapy and Rehabilitation, Faculty of Health Sciences, Acibadem Mehmet Ali Aydinlar University, Atasehir, 34752 Istanbul, Türkiye; mehrad.mohammadzadeh@bahcesehir.edu.tr; 2 Department of Physiotherapy and Rehabilitation, Institute of Graduate Studies, Bahçeşehir University, Beşiktaş, 34353 Istanbul, Türkiye

**Keywords:** cross-sectional studies, musculoskeletal pain, physical activity, screen time, students

## Abstract

**Highlights:**

**What are the main findings?**

- Daily screen time, sitting time, and physical activity were associated with musculoskeletal pain-site count in university students.
- Sitting time was associated with lower back, shoulder, and upper back pain, while screen time was mainly associated with neck pain.

**What are the implications of the main findings?**

- Musculoskeletal pain in university students should be considered within a broader movement-behavior framework rather than attributed to screen exposure alone.
- University-based screening and health education may benefit from considering screen-use habits, sitting time, physical activity, and pain distribution together.

**Abstract:**

**Background/Objectives**: This cross-sectional study examined whether daily screen time, sitting time, and physical activity were associated with musculoskeletal pain-site count among university students. **Methods**: A total of 255 students aged 18–25 years were included. Musculoskeletal pain during the previous 12 months was assessed in nine anatomical regions using the Extended Nordic Musculoskeletal Questionnaire. The primary outcome was pain-site count. Poisson regression with robust standard errors was used for the primary analysis, and region-specific outcomes were examined using multivariable logistic regression. **Results**: Multisite pain was reported by 81 students (31.8%). The most common pain regions were the neck (29.0%), lower back (25.9%), and shoulder (22.7%). In the adjusted Poisson model, each additional hour of daily screen time was associated with a 10% higher pain-site count (IRR = 1.10, 95% CI: 1.04–1.15), and each additional hour of sitting time was associated with a 7% higher pain-site count (IRR = 1.07, 95% CI: 1.03–1.11). Higher physical activity was associated with a lower pain-site count (IRR = 0.93 per 1000 MET-min/week, 95% CI: 0.88–0.98). Female sex and younger age were also associated with higher pain-site count. In region-specific analyses, screen time was associated with neck pain, whereas sitting time was associated with lower back, shoulder, and upper back pain. **Conclusions**: Musculoskeletal pain burden in university students was associated with multiple movement-related behaviors. These findings may inform screening and future longitudinal or intervention studies addressing screen use, sitting time, and physical activity.

## 1. Introduction

University students are increasingly exposed to screen-based behaviors through academic activities, digital learning, social communication, and leisure pursuits. Although screen time frequently occurs in seated postures, it should not be considered synonymous with sitting time, sedentary behavior, or physical inactivity. Screen time refers to time spent using screen-based devices, whereas sitting time reflects time accumulated in a seated posture, regardless of device use. Sedentary behavior is defined by low energy expenditure while sitting, reclining, or lying, whereas physical inactivity refers to not achieving recommended levels of moderate-to-vigorous physical activity. These constructs are therefore related but conceptually distinct [1,2]. Current guidelines emphasize both reducing sedentary time and increasing physical activity, highlighting the importance of examining these behavioral exposures separately [2]. However, their combined relevance to musculoskeletal pain burden among university students remains insufficiently defined [3,4].

Prolonged screen-based device use may be associated with musculoskeletal symptoms through sustained sitting, limited postural variation, forward head posture, repetitive upper-limb activity, and insufficient movement breaks [5,6]. In addition, sedentary behavior has been associated with musculoskeletal pain in both occupational and non-occupational contexts, supporting the need to distinguish sitting-related exposure from screen exposure alone [7]. Much of the previous student literature has focused on smartphone overuse, problematic device use, or pain in selected anatomical regions [8,9]. However, contemporary screen exposure often involves multiple devices and both academic and non-academic purposes. Therefore, examining daily screen time together with sitting time and physical activity may better capture the broader movement-behavior context of musculoskeletal pain in this population.

Musculoskeletal pain is commonly assessed by anatomical region, yet this approach may underestimate the broader burden of symptoms when pain occurs across multiple body sites. Pain-site count may provide complementary information to region-specific outcomes by reflecting the distribution of musculoskeletal symptoms across the body [10,11]. This is relevant for university students because multisite pain may indicate a broader symptom burden than isolated regional pain and may help identify students who could benefit from early screening and health education. Nevertheless, few studies have examined screen time, sitting time, and physical activity within the same analytical framework in relation to multisite musculoskeletal pain among young university students [11].

Therefore, this study aimed to examine whether approximate total daily screen time, sitting time, and physical activity were independently associated with the number of musculoskeletal pain sites among university students aged 18–25 years, and secondarily with selected region-specific pain outcomes. Based on previous literature, we hypothesized that higher daily screen time and longer sitting time would be associated with a higher musculoskeletal pain-site count, whereas higher physical activity would be associated with a lower pain-site count.

## 2. Materials and Methods

### 2.1. Study Design, Setting, and Data Collection

This cross-sectional, questionnaire-based study was conducted at Acibadem Mehmet Ali Aydinlar University, Istanbul, Türkiye, between February and December 2024. The study examined the associations of screen time, sitting time, and physical activity with musculoskeletal pain-site count among university students and was reported in accordance with the STROBE recommendations for observational studies [12]. Ethical approval was obtained from the Acibadem Mehmet Ali Aydinlar University Medical Research Evaluation Committee (ATADEK; decision no: 2024-1/15, 25 January 2024), and all procedures were conducted in accordance with the Declaration of Helsinki. Data were collected via a self-administered online survey distributed through institutional e-mail announcements, with two reminder e-mails sent during recruitment. Participants provided electronic informed consent before completing the questionnaire and were informed that participation was voluntary and responses would remain anonymous and confidential.

### 2.2. Participants

The target population comprised university students aged 18–25 years. Students were eligible if they were enrolled at the university, volunteered to participate, provided electronic informed consent, and reported screen-based device use for at least 2 h per day. This threshold was used to ensure that participants had a minimum level of regular screen exposure relevant to the study question, while retaining variability in screen-use duration across the sample. Participants were excluded if they reported congenital musculoskeletal deformity, musculoskeletal trauma within the previous 6 months, previous musculoskeletal surgery, neurological disease, or incomplete questionnaire data.

### 2.3. Outcome Measures

The primary outcome was the number of musculoskeletal pain sites reported during the previous 12 months. Musculoskeletal pain was assessed using the Extended Nordic Musculoskeletal Questionnaire (NMQ-E) [13,14], which evaluates symptoms in nine anatomical regions: neck, shoulder, upper back, elbow, wrist/hand, lower back, hip/thigh, knee, and ankle/foot. For each participant, the number of painful regions was summed to obtain a count variable ranging from 0 to 9.

For descriptive analyses, pain-site burden was categorized as no pain, single-site pain, and multisite pain, with multisite pain defined as pain in two or more anatomical regions. Participants were also grouped as having 0–1 or ≥2 pain sites to compare those without multisite pain with those with multisite pain. Region-specific pain outcomes were coded as binary variables based on the presence or absence of pain in each anatomical region during the previous 12 months.

Screen use was assessed using a structured screen-based device use form adapted from Khalili-Mahani et al. [15]. The form recorded approximate average daily total screen time, smartphone use duration, device-related screen use, and purpose-specific screen use. Daily total screen time was calculated as the participant’s reported overall daily duration of screen-based device use and included both academic and non-academic screen exposure. Because students may use multiple devices within the same day and may use screens while sitting, standing, or reclining, total screen time and sitting time were treated as separate behavioral exposures and were not summed. Device-related and purpose-specific screen-use characteristics were rated on 1–5 ordinal scales, where 1 indicated “never” and 5 indicated “always”; higher scores indicated more frequent use. The adapted form was used to characterize screen-use patterns in this sample but has not been formally validated in Turkish university students.

Physical activity and sitting time were assessed using the International Physical Activity Questionnaire–Short Form (IPAQ-SF) [16,17]. Total physical activity was calculated as MET-min/week according to standard IPAQ scoring procedures, using 3.3 METs for walking, 4.0 METs for moderate-intensity activity, and 8.0 METs for vigorous-intensity activity [18,19]. IPAQ total physical activity represents intensity-weighted activity volume and should not be interpreted as hours/day [19]. Participants were categorized as having low/inactive, moderate, or high physical activity according to IPAQ scoring criteria [19,20,21,22]. Sitting time was expressed as hours per day and analyzed as a continuous variable.

### 2.4. Sample Size

The sample size was calculated a priori using G*Power version 3.1 (Heinrich Heine University Düsseldorf, Düsseldorf, Germany). Because the primary outcome was the number of musculoskeletal pain sites, the calculation was informed by a previous study examining multisite musculoskeletal pain among young students entering working life [23]. Using a linear multiple regression model for R^2^ increase, with three tested predictors, six total predictors, f^2^ = 0.048, 80% power, and a two-sided α level of 0.05, the minimum required sample size was 232 participants. To account for potential non-response and incomplete or unusable questionnaires, the recruitment target was increased by approximately 30% to 302 students. The final analytic sample included 255 participants, exceeding the minimum required sample size.

### 2.5. Statistical Analysis

Statistical analyses were performed using IBM SPSS Statistics for Windows, version 23.0 (IBM Corp., Armonk, NY, USA). Statistical significance was set at *p* < 0.05. All analyses were conducted using the complete-case analytic sample for the relevant model.

Continuous variables were assessed for normality using histograms, Q–Q plots, and the Shapiro–Wilk test. Normally distributed variables were summarized as mean ± standard deviation, non-normally distributed variables as median and interquartile range, and categorical variables as frequency and percentage. Participant characteristics were compared according to pain-site burden, defined as 0–1 versus ≥2 painful anatomical regions during the previous 12 months. Continuous variables were compared using the independent samples *t*-test or Mann–Whitney U test, as appropriate, and categorical variables using the chi-square test.

The number of musculoskeletal pain sites reported during the previous 12 months was analyzed as the primary count outcome using Poisson regression with robust standard errors. Incidence rate ratios and 95% confidence intervals were reported. The primary multivariable model included daily total screen time, sitting time, total physical activity expressed per 1000 MET-min/week, age, sex, and body mass index. To improve transparency, unadjusted models were also fitted for each predictor and are presented alongside the adjusted estimates in the supplementary material.

Model diagnostics were conducted for the primary count model, following recommendations for count-data modelling in health research [24]. The mean and variance of the pain-site count outcome were examined, and model dispersion was evaluated using Pearson and deviance dispersion statistics. The observed number of zero counts was also compared with the number expected under a Poisson distribution. A negative binomial regression model was fitted as a sensitivity analysis to assess whether allowing for extra-Poisson variation improved model fit. Multicollinearity was evaluated by examining the correlation between daily total screen time and sitting time and by calculating variance inflation factors for the predictors included in the primary multivariable model.

Secondary analyses examined region-specific pain outcomes using multivariable logistic regression. These models were fitted only for anatomical regions with at least 30 pain events during the previous 12 months. Odds ratios and 95% confidence intervals were reported. All logistic regression models were adjusted for age, sex, body mass index, daily total screen time, sitting time, and total physical activity. Because multiple region-specific outcomes were examined, these analyses were considered secondary and exploratory and were not adjusted for multiple comparisons.

To address the difference between the 12-month pain recall period and the more recent behavioral exposures, exploratory sensitivity analyses were conducted using pain-site counts derived from symptoms reported during the previous 4 weeks and symptoms reported today. These models used the same covariates as the primary count model. The 12-month pain-site count remained the primary outcome.

## 3. Results

### 3.1. Participant Flow and Characteristics

**Figure 1:**
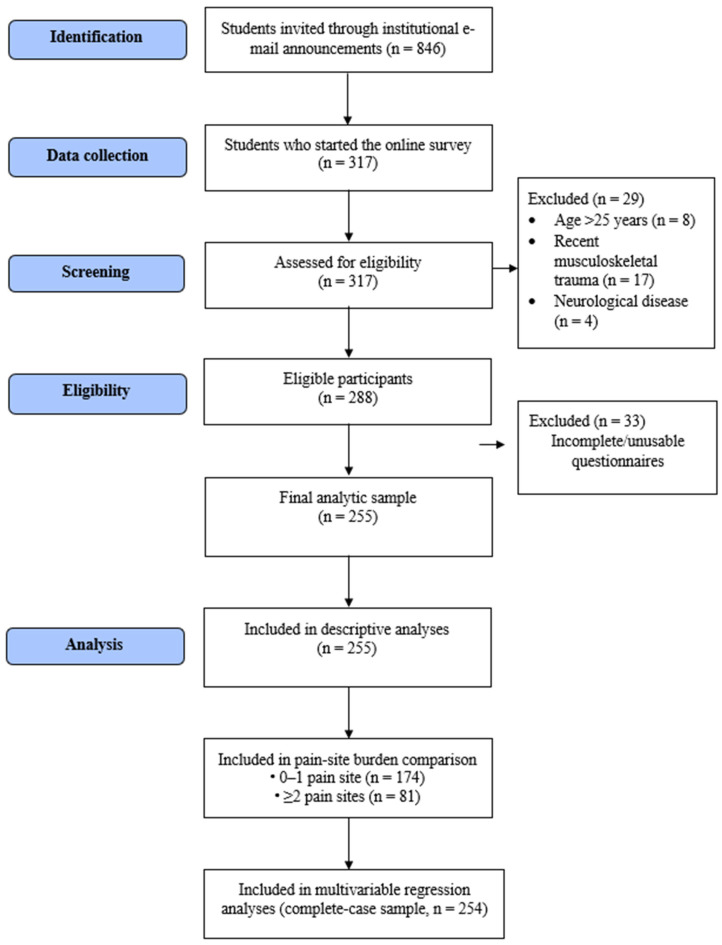
Flow diagram of participant recruitment, eligibility assessment, and inclusion in the final analytic sample.

Of the 846 students invited, 317 initiated the online survey. After eligibility screening, 29 students were excluded because of age >25 years (*n* = 8), recent musculoskeletal trauma (*n* = 17), or neurological disease (*n* = 4). An additional 33 questionnaires were excluded because of incomplete or unusable data. The final analytic sample included 255 participants (Figure 1). Multivariable regression analyses were conducted using the complete-case sample (*n* = 254), as IPAQ total physical activity data were missing for one participant.

The mean age of the participants was 21.2 ± 1.6 years, and 188 participants were female (73.7%). Overall, 174 participants had 0–1 pain site, and 81 had pain in ≥2 anatomical regions during the previous 12 months. Compared with participants with 0–1 pain site, those with ≥2 pain sites were younger, more frequently female, had lower BMI, longer daily total screen time, longer sitting time, and lower total physical activity. The distribution of physical activity categories also differed between groups, with a higher proportion of low/inactive participants among those with ≥2 pain sites (Table 1).

### 3.2. Musculoskeletal Pain Distribution and Screen-Use Characteristics

During the previous 12 months, 75 participants reported no musculoskeletal pain (29.4%), 99 reported single-site pain (38.8%), and 81 reported multisite pain (≥2 anatomical regions; 31.8%). The most frequently reported pain regions were the neck (29.0%), lower back (25.9%), shoulder (22.7%), and upper back (15.3%) (Table 2).

Screen-use characteristics are presented in Table 3. Educational or information-seeking use, video/movie watching, and entertainment-related use were among the highest-rated screen-use purposes. Smartphone use had the highest device-related screen-use score, whereas desktop computer, game console, and television use had lower scores.

### 3.3. Model Diagnostics and Multicollinearity Assessment

For the primary 12-month pain-site count outcome, the mean count was 1.18 and the variance was 1.14. The observed number of zero counts was 75, compared with 78.0 expected under a Poisson distribution. The Pearson and deviance dispersion statistics were 0.86 and 0.91, respectively, indicating no evidence of overdispersion and supporting the adequacy of the Poisson model. In the negative binomial sensitivity model, the overdispersion parameter approached zero, and the model did not indicate improved fit compared with the Poisson model. Therefore, Poisson regression with robust standard errors was retained as the primary count model.

The correlation between daily total screen time and sitting time was low (*r* = 0.15). Variance inflation factors ranged from 1.04 to 1.13 across the predictors included in the primary multivariable model, indicating no evidence of problematic multicollinearity (Supplementary Table S1).

### 3.4. Factors Associated with Musculoskeletal Pain-Site Count

In the primary multivariable Poisson regression model with robust standard errors, daily total screen time, sitting time, total physical activity, female sex, and age were associated with the number of musculoskeletal pain sites. IRRs were interpreted as adjusted count ratios for the number of painful anatomical regions. Each additional hour of daily total screen time was associated with a 10% higher pain-site count (IRR = 1.10, 95% CI: 1.04–1.15, *p* < 0.001). Each additional hour of sitting time was associated with a 7% higher pain-site count (IRR = 1.07, 95% CI: 1.03–1.11, *p* < 0.001). Higher total physical activity was associated with a lower pain-site count (IRR = 0.93 per 1000 MET-min/week, 95% CI: 0.88–0.98, *p* = 0.008). Female sex was associated with a higher pain-site count (IRR = 1.56, 95% CI: 1.20–2.02, *p* = 0.001), whereas age was inversely associated with pain-site count (IRR = 0.93, 95% CI: 0.87–0.99, *p* = 0.024). BMI was not significantly associated with pain-site count (Table 4). Unadjusted and adjusted estimates are presented in Supplementary Table S2.

### 3.5. Region-Specific Associations with Musculoskeletal Pain

Region-specific logistic regression analyses were conducted as secondary exploratory analyses for anatomical regions with at least 30 pain events during the previous 12 months using the complete-case sample (*n* = 254). Daily total screen time was associated with higher odds of neck pain (OR = 1.163, 95% CI: 1.027–1.318, *p* = 0.017), and total physical activity was inversely associated with neck pain (OR = 0.835 per 1000 MET-min/week, 95% CI: 0.709–0.984, *p* = 0.032). Age was also inversely associated with neck pain (OR = 0.791, 95% CI: 0.648–0.965, *p* = 0.021). Sitting time was not significantly associated with neck pain after adjustment (OR = 1.070, 95% CI: 0.960–1.192, *p* = 0.225).

Sitting time was associated with higher odds of lower back pain (OR = 1.192, 95% CI: 1.064–1.335, *p* = 0.002), shoulder pain (OR = 1.158, 95% CI: 1.030–1.301, *p* = 0.014), and upper back pain (OR = 1.210, 95% CI: 1.055–1.389, *p* = 0.006). Age was inversely associated with lower back pain (OR = 0.816, 95% CI: 0.668–0.997, *p* = 0.046). Other covariates were not significantly associated with the selected region-specific pain outcomes (Table 5).

### 3.6. Sensitivity Analyses Using Alternative Pain Time Windows

Exploratory sensitivity analyses were conducted using pain-site counts derived from symptoms reported during the previous 4 weeks and pain reported today. In the previous 4-week model, sitting time, female sex, and age were associated with pain-site count, whereas daily total screen time and total physical activity were not statistically significant. In the model based on pain reported today, daily total screen time, sitting time, total physical activity, and age were associated with pain-site count. These sensitivity analyses are presented in Supplementary Table S3.

## 4. Discussion

This study examined the associations of daily total screen time, sitting time, and physical activity with 12-month musculoskeletal pain-site count among university students. In the primary adjusted model, higher daily screen time and longer sitting time were associated with a greater number of painful anatomical regions, whereas higher physical activity was associated with a lower pain-site count. Female sex and younger age were also associated with higher pain-site count. In secondary exploratory region-specific analyses, daily total screen time was associated with neck pain, while sitting time was associated with lower back, shoulder, and upper back pain. Overall, these findings support the relevance of examining musculoskeletal pain in university students within a broader movement-behavior framework rather than in relation to screen exposure alone.

Nearly one-third of the students reported multisite musculoskeletal pain, with the neck, lower back, and shoulder being the most frequently affected regions. This distribution is consistent with previous studies reporting frequent spinal and upper-quarter symptoms among university students and young adults exposed to academic, digital, and sedentary activities [8,9,25,26]. The present study extends this literature by using pain-site count as the primary outcome. Although region-specific pain outcomes remain clinically informative, pain-site count provides a complementary indicator of symptom distribution across multiple anatomical regions [10,11]. This approach may be particularly relevant in student health settings, where identifying students with more distributed musculoskeletal symptoms may support early screening and health education.

The association between daily total screen time and pain-site count suggests that overall screen exposure may be relevant to musculoskeletal symptom burden. The exploratory association between screen time and neck pain is also consistent with previous evidence linking screen-based behaviors, particularly smartphone-related exposure, with neck and upper-body symptoms [6,25,26,27]. However, total screen time is a broad exposure and does not distinguish device type, posture, workstation characteristics, task demands, break frequency, or uninterrupted duration of use. Therefore, these findings should be interpreted as indicating an association with overall screen exposure rather than identifying a specific ergonomic or behavioral mechanism.

Sitting time was also associated with pain-site count and, in exploratory region-specific models, with lower back, shoulder, and upper back pain. These findings highlight the importance of evaluating sitting time separately from screen time. Although these constructs may overlap, they are not interchangeable: screen use may occur while sitting, standing, or reclining, whereas sitting time may include both screen-based and non-screen activities. Previous evidence has linked sedentary behavior with musculoskeletal pain across occupational and non-occupational settings [7], and systematic reviews have reported associations between sedentary behavior and low back pain or neck pain [6,28]. In the present study, the association of sitting time with lower back, shoulder, and upper back pain suggests that prolonged sitting may be relevant to a broader spinal and upper-quarter symptom pattern in university students.

Higher physical activity was associated with lower pain-site count and, in exploratory region-specific analyses, with lower odds of neck pain. This finding is consistent with current recommendations emphasizing both regular physical activity and reduction of sedentary time [2]. Nevertheless, the direction of this association cannot be determined from the cross-sectional design. Lower physical activity may contribute to greater musculoskeletal pain burden, but students experiencing pain may also reduce their activity because of discomfort, avoidance, or functional limitation [29]. This interpretation is further complicated by the difference between the 12-month pain recall period and the current or recent nature of the behavioral exposures. Sensitivity analyses using shorter pain recall windows were conducted to explore this issue, but longitudinal studies are required to clarify temporal relationships.

Female sex was associated with higher pain-site count in the primary model, and several region-specific estimates for female sex were elevated, although not consistently statistically significant. Sex-related differences in musculoskeletal pain prevalence and reporting have been described in previous studies involving students and young adults [25,26]. In the present sample, the high proportion of female participants should be considered when interpreting generalizability. Although sex was included as an adjustment variable, the study was not designed to examine sex-specific pathways or interactions. Future studies with more balanced samples should evaluate whether the associations between movement-related behaviors and multisite pain differ by sex.

The findings have potential implications for university-based screening and health education. They suggest that musculoskeletal pain burden in students may be better addressed by considering screen-use habits, sitting behavior, physical activity, and pain distribution together rather than focusing on screen exposure alone. For students with multisite pain, assessment of these movement-related behaviors and study-related ergonomic conditions may help identify candidate behavioral and ergonomic factors for future longitudinal and intervention studies. However, the present findings should not be interpreted as evidence that modifying screen time, sitting time, or physical activity will reduce musculoskeletal pain.

### Limitations and Future Directions

This study has several strengths, including the simultaneous assessment of screen time, sitting time, and physical activity, the use of pain-site count as the primary outcome, and the inclusion of additional model diagnostics and sensitivity analyses. Nevertheless, several limitations should be acknowledged. The cross-sectional design precludes conclusions regarding temporality or causality. Moreover, the primary pain outcome referred to symptoms experienced during the previous 12 months, whereas screen time, sitting time, and physical activity reflected current or typical recent behavior. Thus, these behavioral exposures may not fully represent patterns of exposure across the entire pain recall period.

All behavioral exposures and musculoskeletal pain outcomes were self-reported; therefore, recall bias and reporting error cannot be excluded. Daily total screen time and sitting time were analyzed as separate exposures and were not summed because these constructs may overlap. Similarly, IPAQ total physical activity was expressed as MET-min/week, an intensity-weighted activity volume, and should not be interpreted as hours per day. In addition, total screen time did not capture device-specific duration, posture, workstation setup, visual ergonomics, break frequency, or uninterrupted sitting bouts. These factors may be important for clarifying the relationships between digital behaviors, sedentary exposure, postural loading, and musculoskeletal symptoms.

Although pain-site count provides a useful indicator of the anatomical distribution of musculoskeletal symptoms, it does not capture pain intensity, frequency, duration, disability, healthcare-seeking behavior, or symptom persistence. Future studies should therefore combine pain-site count with measures of pain severity, functional limitation, and clinical relevance. Potential confounders, including sleep quality, psychological distress, academic workload, previous pain history, and ergonomic environment, were not assessed and may have influenced both movement-related behaviors and musculoskeletal pain [30]. Residual confounding should therefore be considered when interpreting the observed associations.

The study was conducted at a single university and included a high proportion of female students, which may limit the generalizability of the findings. Recruitment through institutional e-mail announcements may also have introduced self-selection bias, and detailed faculty, academic-year, and field-specific representativeness could not be fully established from the available data. Future research should use longitudinal designs and, where possible, objective or repeated measures such as accelerometry, posture monitoring, device-use logs, and ecological momentary assessment. Such approaches may help clarify temporal relationships and distinguish total screen exposure from sitting accumulation, device-specific behavior, and postural loading. Intervention studies are also needed to determine whether strategies targeting candidate movement-related and ergonomic factors can reduce multisite musculoskeletal pain among university students.

## 5. Conclusions

In this cross-sectional study of university students, daily total screen time, sitting time, and total physical activity were associated with 12-month musculoskeletal pain-site count after adjustment for age, sex, and BMI. Higher daily screen time and longer sitting time were associated with a greater number of painful anatomical regions, whereas higher physical activity was associated with a lower pain-site count. These findings suggest that musculoskeletal pain burden in university students may be better understood within a broader movement-behavior framework rather than in relation to screen exposure alone.

In secondary exploratory analyses, daily total screen time was associated with neck pain, whereas sitting time was associated with lower back, shoulder, and upper back pain. Given the cross-sectional design and the exploratory nature of the region-specific analyses, these findings should be interpreted cautiously. Future longitudinal and intervention studies are needed to clarify temporal relationships and to evaluate whether candidate movement-related and ergonomic factors can reduce multisite musculoskeletal pain in university students.

## Figures and Tables

**Table 1:** Participant characteristics according to musculoskeletal pain-site burden.

Variable	Total Sample (*n* = 255)	0–1 Pain Site (*n* = 174)	≥2 Pain Sites (*n* = 81)	*p*-Value
Age, years	21.2 ± 1.6	21.4 ± 1.6	20.8 ± 1.4	0.011
Sex				0.001
Female	188 (73.7)	117 (67.2)	71 (87.7)	
Male	67 (26.3)	57 (32.8)	10 (12.3)	
BMI, kg/m^2^	22.4 ± 3.4	22.9 ± 3.5	21.5 ± 3.0	0.004
Daily total screen time, h/day	6.6 ± 2.4	6.2 ± 2.2	7.3 ± 2.6	0.001
Sitting time, h/day	8.0 ± 2.7	7.5 ± 2.5	9.0 ± 2.8	<0.001
**IPAQ-based variables**	**Total Sample** **(*n* = 254)**	**0–1 Pain Site** **(*n* = 174)**	**≥2 Pain Sites** **(*n* = 80)**	***p*** **-Value**
IPAQ total physical activity, MET-min/week	1800 (750–3300)	2100 (900–3700)	1358 (600–2600)	0.028
**Physical activity category**				0.003
Low/inactive	76 (29.9)	43 (24.7)	33 (41.3)	
Moderate	115 (45.3)	79 (45.4)	36 (45.0)	
High	63 (24.8)	52 (29.9)	11 (13.7)	

Values are presented as mean ± SD, median (IQR), or *n* (%). Pain-site burden was defined as 0–1 versus ≥2 painful anatomical regions during the previous 12 months. IPAQ-based variables were analyzed using available data (total sample, *n* = 254; 0–1 pain-site group, *n* = 174; ≥2 pain-sites group, *n* = 80). Continuous variables were compared using the independent samples *t*-test or Mann–Whitney U test, as appropriate, and categorical variables using the chi-square test. Daily total screen time and sitting time are not mutually exclusive. BMI, body mass index; IPAQ, International Physical Activity Questionnaire; IQR, interquartile range; SD, standard deviation.

**Table 2:** Pain-site burden and anatomical distribution of musculoskeletal pain during the previous 12 months.

**A. Pain-site burden**		
**Pain-site burden category**		**Total sample, *n*/N (%)**
No pain		75/255 (29.4)
Single-site pain		99/255 (38.8)
Multisite pain, ≥2 sites		81/255 (31.8)
**B. Number of pain sites**		
**Number of pain sites**		***n*** **/N (%)**
0 pain site		75/255 (29.4)
1 pain site		99/255 (38.8)
2 pain sites		50/255 (19.6)
3 pain sites		24/255 (9.4)
4 pain sites		5/255 (2.0)
5 pain sites		2/255 (0.8)
6–9 pain sites		0/255 (0.0)
**C. Region-specific pain during the previous 12 months**		
**Pain region**	**Total sample, *n*/N (%)**	**Participants with any pain, *n*/N (%)**
Neck	74/255 (29.0)	74/180 (41.1)
Lower back	66/255 (25.9)	66/180 (36.7)
Shoulder	58/255 (22.7)	58/180 (32.2)
Upper back	39/255 (15.3)	39/180 (21.7)
Wrist/hand	18/255 (7.1)	18/180 (10.0)
Knee	18/255 (7.1)	18/180 (10.0)
Ankle/foot	13/255 (5.1)	13/180 (7.2)
Hip/thigh	10/255 (3.9)	10/180 (5.6)
Elbow	5/255 (2.0)	5/180 (2.8)

Pain-site burden categories and pain-site counts were calculated using the total analytic sample (*n* = 255). Region-specific percentages are presented for both the total sample and participants reporting at least one musculoskeletal pain site during the previous 12 months (*n* = 180). Region-specific pain categories were not mutually exclusive.

**Table 3:** Screen-use purpose and device-related screen-use characteristics of the study sample.

Variable	Median (IQR)
**Screen-use purpose score**	
Educational use/information seeking	4.0 (3.0–5.0)
Video/movie watching	4.0 (3.0–5.0)
Social networking	3.0 (2.0–4.0)
Entertainment	4.0 (3.0–4.0)
News/e-reading	3.0 (2.0–4.0)
Rest/leisure	3.0 (2.0–4.0)
Online shopping	3.0 (2.0–4.0)
SMS/e-mail communication	2.0 (1.0–3.0)
Professional networking/work-related use	2.0 (1.0–3.0)
Gaming	2.0 (1.0–3.0)
**Device-related screen-use score**	
Smartphone	4.0 (4.0–5.0)
Laptop computer	2.0 (1.0–3.0)
E-reader	1.0 (1.0–2.0)
Tablet	2.0 (1.0–3.0)
Desktop computer	1.0 (1.0–2.0)
Game console	1.0 (1.0–1.0)
Television	1.0 (1.0–2.0)

Values are presented as median (IQR). Screen-use purpose and device-related scores were rated on a 1–5 ordinal scale, with higher scores indicating greater frequency (*n* = 255).

**Table 4:** Multivariable Poisson regression with robust standard errors for the number of musculoskeletal pain sites. **Outcome:** 12-month musculoskeletal pain-site count. **Complete-case sample:** *n* = 254.

Predictor	IRR	95% CI	*p*-Value
Daily total screen time, per 1 h/day	1.10	1.04–1.15	<0.001
Sitting time, per 1 h/day	1.07	1.03–1.11	<0.001
IPAQ total physical activity, per 1000 MET-min/week	0.93	0.88–0.98	0.008
Female sex	1.56	1.20–2.02	0.001
Age, years	0.93	0.87–0.99	0.024
BMI, kg/m^2^	0.98	0.95–1.01	0.236

IRRs were estimated using Poisson regression with robust standard errors and are interpreted as adjusted count ratios for the number of painful anatomical regions. The model included age, sex, BMI, daily total screen time, sitting time, and IPAQ total physical activity. The analysis used the complete-case sample (*n* = 254). BMI, body mass index; CI, confidence interval; IPAQ, International Physical Activity Questionnaire; IRR, incidence rate ratio.

**Table 5:** Multivariable logistic regression models for selected region-specific pain outcomes.

Pain Region	Events/N	Predictor	Adjusted OR	95% CI	*p*-Value
Neck	74/254	Daily total screen time, per 1 h/day	1.163	1.027–1.318	0.017
		Sitting time, per 1 h/day	1.070	0.960–1.192	0.225
		IPAQ total physical activity, per 1000 MET-min/week	0.835	0.709–0.984	0.032
		Female sex	2.150	0.974–4.747	0.058
		Age, years	0.791	0.648–0.965	0.021
		BMI, kg/m^2^	0.986	0.902–1.079	0.764
Lower back	66/254	Daily total screen time, per 1 h/day	1.103	0.972–1.253	0.129
		Sitting time, per 1 h/day	1.192	1.064–1.335	0.002
		IPAQ total physical activity, per 1000 MET-min/week	0.995	0.870–1.138	0.940
		Female sex	1.657	0.761–3.609	0.204
		Age, years	0.816	0.668–0.997	0.046
		BMI, kg/m^2^	0.949	0.866–1.041	0.270
Shoulder	58/254	Daily total screen time, per 1 h/day	1.028	0.903–1.170	0.679
		Sitting time, per 1 h/day	1.158	1.030–1.301	0.014
		IPAQ total physical activity, per 1000 MET-min/week	0.906	0.770–1.066	0.235
		Female sex	2.148	0.913–5.057	0.080
		Age, years	0.902	0.736–1.105	0.319
		BMI, kg/m^2^	0.933	0.848–1.027	0.157
Upper back	39/254	Daily total screen time, per 1 h/day	0.918	0.790–1.067	0.266
		Sitting time, per 1 h/day	1.210	1.055–1.389	0.006
		IPAQ total physical activity, per 1000 MET-min/week	0.880	0.718–1.078	0.216
		Female sex	2.962	0.963–9.109	0.058
		Age, years	0.923	0.729–1.169	0.508
		BMI, kg/m^2^	0.942	0.845–1.051	0.285

ORs were estimated using multivariable logistic regression. Models were fitted for anatomical regions with ≥30 pain events during the previous 12 months and adjusted for age, sex, BMI, daily total screen time, sitting time, and IPAQ total physical activity. Events/N refers to the complete-case sample used in the corresponding models (*n* = 254). Region-specific analyses were secondary/exploratory and were not adjusted for multiple comparisons. BMI, body mass index; CI, confidence interval; IPAQ, International Physical Activity Questionnaire; OR, odds ratio.

## Data Availability

The data presented in this study are available on request from the corresponding author. The data are not publicly available due to privacy restrictions.

## Supplementary Materials

The following supporting information can be downloaded at: https://www.mdpi.com/article/10.3390/healthcare14142154/s1. The following supplementary tables provide additional statistical diagnostics, unadjusted and adjusted regression estimates, and exploratory sensitivity analyses supporting the main findings. Table S1: Primary count-model diagnostics and multicollinearity assessment; Table S2: Unadjusted and adjusted Poisson regression models for the 12-month musculoskeletal pain-site count; Table S3: Exploratory sensitivity analyses using alternative pain recall windows.

## Abbreviations

The following abbreviations are used in this manuscript:

BMI	Body mass index
CI	Confidence interval
IPAQ	International Physical Activity Questionnaire
IPAQ-SF	International Physical Activity Questionnaire–Short Form
IQR	Interquartile range
IRR	Incidence rate ratio
MET	Metabolic equivalent of task
NMQ-E	Extended Nordic Musculoskeletal Questionnaire
OR	Odds ratio
SD	Standard deviation
STROBE	Strengthening the Reporting of Observational Studies in Epidemiology
VIF	Variance inflation factor
