# Supplementary material for: Screen Time, Sitting Time, Physical Activity, and Multisite Musculoskeletal Pain in University Students: Implications for Preventive Healthcare
Source: Healthcare (Basel). 2026 Jul 17;14(14):2154. doi: 10.3390/healthcare14142154 (PMC13410034; doi:10.3390/healthcare14142154)
Supplement: Supplementary file 1 [file healthcare-14-02154-s001.zip › healthcare-4350513-supplementary.pdf]

Supplementary Materials

The following supplementary tables provide additional statistical diagnostics, unadjusted and adjusted regression estimates, and exploratory sensitivity analyses supporting the main findings.

Table S1. Primary count-model diagnostics and multicollinearity assessment.

**S1A. Diagnostics for the 12-month pain-site count outcome**	
**Diagnostic**	**Value**
Complete-case *n*	254
Mean pain-site count	1.18
Variance pain-site count	1.14
Observed zero count	75
Expected zero count under Poisson distribution	78.0
Pearson dispersion statistic	0.86
Deviance dispersion statistic	0.91
Negative binomial overdispersion parameter	Approached 0
Negative binomial model status	Did not improve fit compared with the Poisson model
**S1B. Correlation and variance inflation factors**	
**Assessment**	**Value**
Correlation between daily total screen time and sitting time	$r = 0.15$
**Predictor**	**VIF**
Daily total screen time	1.08
Sitting time	1.04
IPAQ total physical activity	1.12
Female sex	1.13
Age	1.06
BMI	1.04

Diagnostics refer to the primary Poisson regression model with robust standard errors for the 12-month pain-site count outcome. The analysis used the complete-case sample ($n = 254$). VIFs were calculated for predictors included in the primary multivariable model. BMI, body mass index; IPAQ, International Physical Activity Questionnaire; VIF, variance inflation factor.

Table S2. Unadjusted and adjusted Poisson regression models for the 12-month musculoskeletal pain-site count.

Predictor	Unadjusted IRR	95% CI	*p*-Value	Adjusted IRR	95% CI	*p*-Value
Daily total screen time, per 1 h/day	1.12	1.07–1.18	<0.001	1.10	1.04–1.15	<0.001
Sitting time, per 1 h/day	1.08	1.04–1.13	<0.001	1.07	1.03–1.11	<0.001
IPAQ total physical activity, per 1000 MET-min/week	0.92	0.87–0.97	0.004	0.93	0.88–0.98	0.008
Female sex	1.88	1.41–2.51	<0.001	1.56	1.20–2.02	0.001
Age, years	0.88	0.83–0.95	<0.001	0.93	0.87–0.99	0.024
BMI, kg/m^2	0.97	0.94–1.00	0.061	0.98	0.95–1.01	0.236

IRRs were estimated using Poisson regression with robust standard errors and are interpreted as count ratios for the number of painful anatomical regions. Adjusted estimates were obtained from the primary multivariable model including age, sex, BMI, daily total screen time, sitting time, and IPAQ total physical activity. The analysis used the complete-case sample (*n* = 254). BMI, body mass index; CI, confidence interval; IPAQ, International Physical Activity Questionnaire; IRR, incidence rate ratio.

Table S3. Exploratory sensitivity analyses using alternative pain recall windows. **Outcome:** Pain-site count for each recall window. **Complete-case sample:** *n* = 254.

Predictor	12-Month Pain-Site Count, IRR (95% CI), *p*-Value	Previous 4-Week Pain-Site Count, IRR (95% CI), *p*-Value	Pain Reported Today, IRR (95% CI), *p*-Value
Daily total screen time, per 1 h/day	1.10 (1.04–1.15), p<0.001	1.04 (0.97–1.11), p=0.243	1.09 (1.00–1.19), p=0.041
Sitting time, per 1 h/day	1.07 (1.03–1.11), p<0.001	1.09 (1.03–1.15), p=0.001	1.14 (1.06–1.23), p<0.001
IPAQ total physical activity, per 1000 MET-min/week	0.93 (0.88–0.98), p=0.008	0.93 (0.87–1.01), p=0.084	0.89 (0.81–0.98), p=0.013
Female sex	1.56 (1.20–2.02), p=0.001	2.00 (1.42–2.82), p<0.001	1.17 (0.78–1.76), p=0.447
Age, years	0.93 (0.87–0.99), p=0.024	0.88 (0.81–0.97), p=0.007	0.86 (0.78–0.95), p=0.004
BMI, kg/m^2	0.98 (0.95–1.01), p=0.236	0.97 (0.93–1.00), p=0.063	0.99 (0.94–1.04), p=0.697

Values are adjusted IRRs with 95% CIs and *p*-values. IRRs were estimated using Poisson regression with robust standard errors and are interpreted as adjusted count ratios for the number of painful anatomical regions. All models included daily total screen time, sitting time, IPAQ total physical activity, age, sex, and BMI. The 12-month pain-site count was the primary outcome; previous 4-week and pain-reported-today models were exploratory sensitivity analyses. BMI, body mass index; CI, confidence interval; IPAQ, International Physical Activity Questionnaire; IRR, incidence rate ratio; MET, metabolic equivalent of task.
